# Thermal Treatment of Recycled Concrete Fines for Sustainable Cement: Linking Physicochemical Transformations, Reactivity, and Life Cycle Assessment

**DOI:** 10.1021/acsomega.5c09513

**Published:** 2026-01-24

**Authors:** Rui Jing, Jian Li Hao, Han-Mei Chen, Engui Liu

**Affiliations:** a Department of Civil Engineering, 122238 Xi’an Jiaotong-Liverpool University, Suzhou, Jiangsu 215123, China; b School of Architecture, 4591 University of Liverpool, Liverpool L69 7ZN, United Kingdom

## Abstract

Recycled concrete fines (RCF), generated during the demolition and recycling of concrete structures, are a promising secondary raw material for cementitious systems, yet their low reactivity has limited large-scale use. In this study, RCFs were subjected to controlled thermal treatments at 400, 600, and 800 °C to systematically investigate their physicochemical transformations and subsequent influence on the blended cement performance. Characterization revealed temperature-dependent modifications in particle size, surface property, phase assemblage, and morphology, which were directly correlated to hydration kinetics, rheology, and mechanical strength. Among the tested conditions, thermal treatment at 600 °C optimally enhanced the RCF reactivity by decomposing weak hydrates while preserving carbonate phases, leading to improved hydration, workable rheology, and higher compressive strength. A cradle-to-gate life cycle assessment further quantified the environmental trade-offs, showing that moderate activation conditions can balance property enhancement with reduced carbon emissions. This integrated analysis highlights thermal treatment as a practical pathway for valorizing recycled concrete fines in sustainable cementitious systems, advancing circular economy practices and low-carbon construction materials.

## Graphical abstract

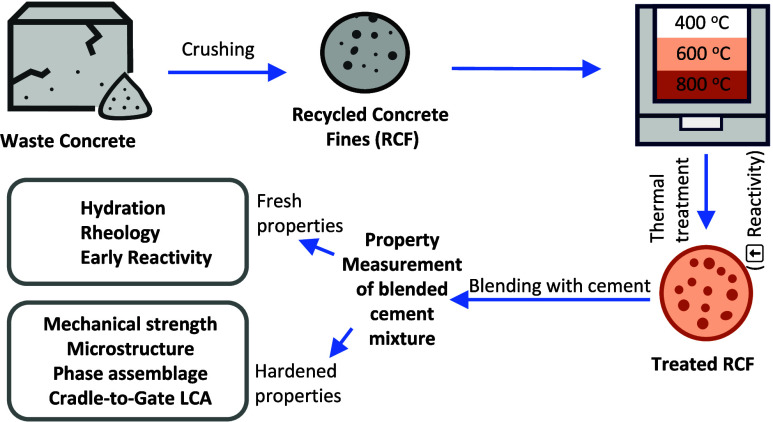

## 1 Introduction

Urbanization and urban regeneration have significantly increased construction activities, leading to a rise in demolition and renovation projects,^1^ which generate large volumes of construction and demolition waste, primarily in the form of concrete.^2,3^ Much of this waste is inadequately treated, contributing to land use issues and environmental pollution.^4–6^ At the same time, cement production remains a major source of carbon emissions, and no full-scale alternatives are expected in the near future, making it a critical obstacle to achieving carbon neutrality. Converting waste concrete into recycled minerals for use in cement offers a promising strategy to reduce landfill burdens and cement consumption, thereby contributing to sustainability goals.^7,8^

Among the components of waste concrete, coarse aggregates and much of the fine aggregates are relatively well recycled and reused in new mixtures. However, the remaining 30–50%, known as recycled concrete fines (RCF), primarily hydrated cement paste and fine mortar particles, remains largely underutilized.^9^ Unlike recycled aggregates, RCF is often discarded due to unfavorable properties that hinder direct reuse. Nevertheless, RCF exhibits potential chemical compatibility with cement owing to its silica-, calcium-, and alumina-rich phases. These compositions allow RCF to participate in hydration through pozzolanic reactions, filler effects, and nucleation promotion.^8,10–13^ When properly processed, RCF can serve as a viable mineral additive, supporting both emission reduction and resource circularity.^14,15^

A widely studied approach is grinding RCF into fine powders for incorporation into cement. Li et al.^11^ reported an activity index of 67–75% (based on 28 days of compressive strength) for processed RCF, attributed to enhanced fineness and particle surface produced by ball milling. Finer RCF particles improve mortar compactness more effectively than coarser particles, while mixed particle sizes also have positive effects.^16^ RCF particles with sizes similar to cement have been shown to promote filler and nucleation efficiency.^17^ However, increasing the dosage can increase drying shrinkage^13^ and internal stresses, ultimately reducing mechanical strength.^18^

Direct use of RCF is limited by its low pozzolanic reactivity, high water demand, and the presence of inert phases. Several modifications have been developed to overcome these challenges. Mechanical grinding enhances surface properties and induces structural changes but only moderately improves reactivity.^13,17^ Carbonation treatment^19^ promotes reactions between CO_2_ and RCF components (e.g., portlandite, C–S–H), forming CaCO_3_ and silica gel that enhance surface properties and reactivity, but requires a controlled CO_2_ environment. Alkaline activation with NaOH or Ca­(OH)_2_ has also been used to increase chemical reactivity,^20,13,21^ though RCF is less reactive than conventional alkali-activated precursors such as fly ash.

While promising, these methods have limitations.^22^ Thermal treatment, similar to cement production but at significantly lower temperatures, offers potential by dehydrating hydration products, altering microstructures, and reactivating cementitious components such as calcium silicates.^23,24^ Wang et al.^25^ identified 450 °C as optimal for inducing dehydration while preserving high-temperature-stable phases like carbonates, which serve as nucleation sites. Other studies^26^ showed that treatment between 600 and 900 °C improves RCF performance in cement systems, though this must be balanced against energy use and cost.^27^ Xu et al.^28^ suggested 600–750 °C as optimal, achieving phase activation without decomposing carbonates at higher temperatures. Zhang et al.^17^ reported increased reactivity after treatment at 800 °C, attributed to decomposition of hydration products and formation of amorphous phases.

Despite these advances, inconsistencies remain regarding the optimal treatment temperature and its influence on RCF performance. This study addresses these gaps by systematically investigating RCF treated at multiple temperatures. RCF, prepared with particle sizes comparable to those of cement, was analyzed before and after treatment and blended into cement for evaluation of hydration behavior, rheology, mechanical strength, and microstructure. To link treatment conditions with environmental impacts, a life cycle assessment (LCA) was conducted. The findings identify treatment conditions that balance performance enhancement with carbon reduction, extending our understanding of RCF as a recycled material and supporting circular economy practices in low-carbon cementitious systems.

## 2 Experiments

### 2.1 Materials

Figure 1 shows the process of obtaining the RCF in this study. Waste concrete was manually hammered, machine-crushed, and sieved through 75 μm mesh to produce initial RCF. The RCF was then thermally treated in a furnace at 400 °C, 600 °C, and 800 °C, with each temperature maintained for 2 h, followed by air cooling. The treated RCFs were mixed with ordinary Portland cement (OPC) and distilled water to prepare blended cement paste specimens.

**Figure 1:**
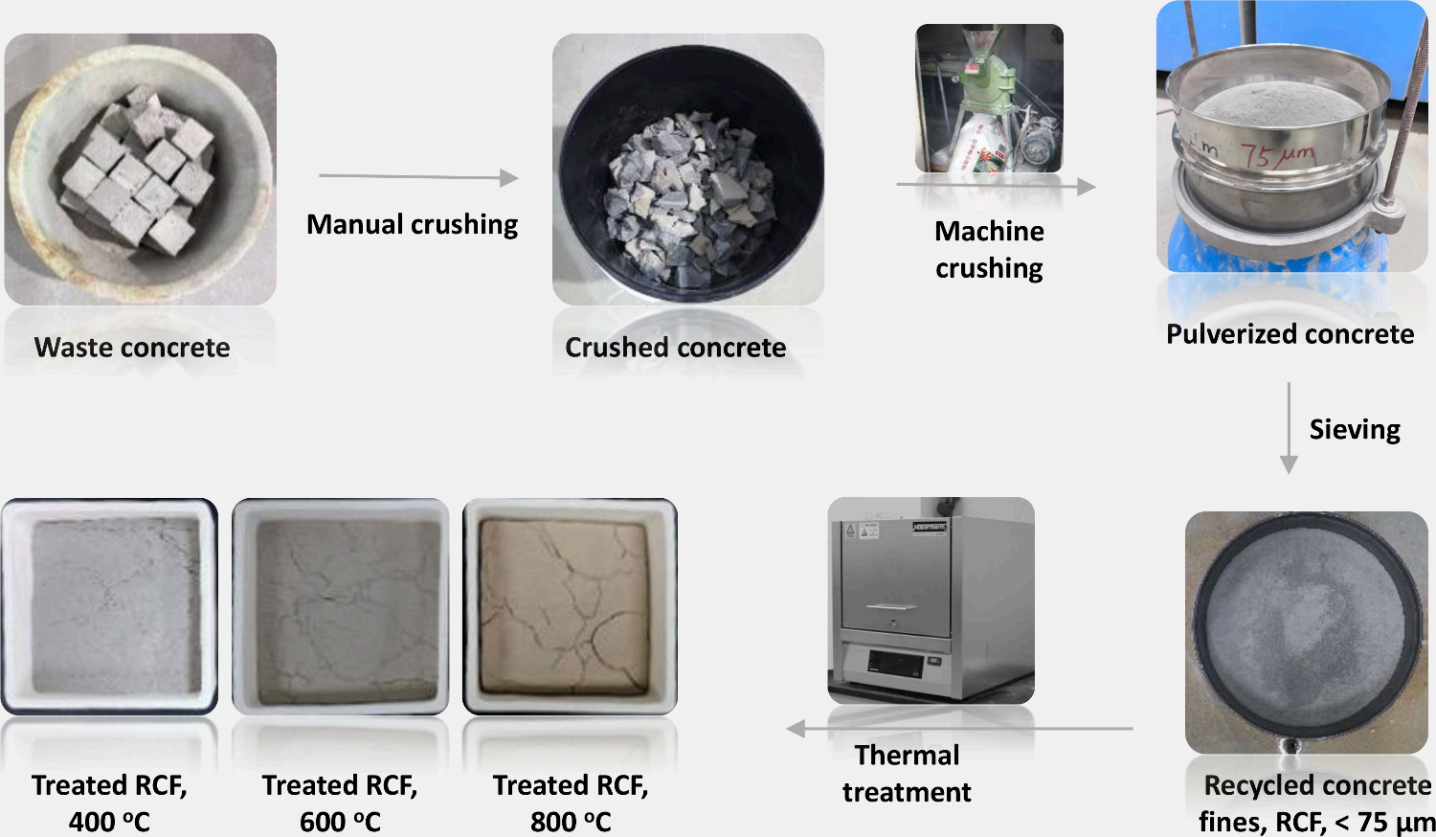
Process for preparing thermally treated RCF at varying temperatures.

### 2.2 Mix Design and Experimental Workflow

This study used various mass dosages of RCF as a cement replacement, maintaining a water-to-binder ratio of 0.40. The experimental design included 8 mixture groups (Table 1), where “OPC” represents pure cement without RCF. The “RCF*x*” indicates the RCF dosage in the blended cement, e.g., “RCF5” refers to 5% RCF and 95% cement by mass. For thermally treated RCF, the dosage was fixed at 10%, e.g., “RCF10-400” refers to a blended cement with 10% RCF treated at 400 °C.

**Table 1:** Mix Proportions of Blended Cement Pastes^a^

mix	OPC	RCF5	RCF10	RCF20	RCF30	RCF10-400	RCF10-600	RCF10-800
cement	100	95	90	80	70	90	90	90
RCF	0	5	10	20	30	10	10	10
water	40	40	40	40	40	40	40	40

a By mass percentage of cement.

Figure 2 shows the overall experimental procedure of the study, including the characterization of RCF and blended cement containing RCF, followed by life cycle assessment of blended cements. The preparation of cement mixtures followed the EN 196-1:2016 guidelines.^29^ OPC and RCF were dry-mixed at 220 rpm for 30 s. Water was then added, and mixing was continued at the same speed for an additional 30 s. The speed was increased to 440 rpm, and the mixture was mixed for 30 s to complete. The mixture was promptly transferred to 40 mm × 40 mm × 40 mm molds, manually vibrated, and compacted. Specimens were cured in a laboratory environment (22 ± 2 °C and 60% relative humidity) for 24 h before demolding, followed by continued curing for specified durations.

**Figure 2:**
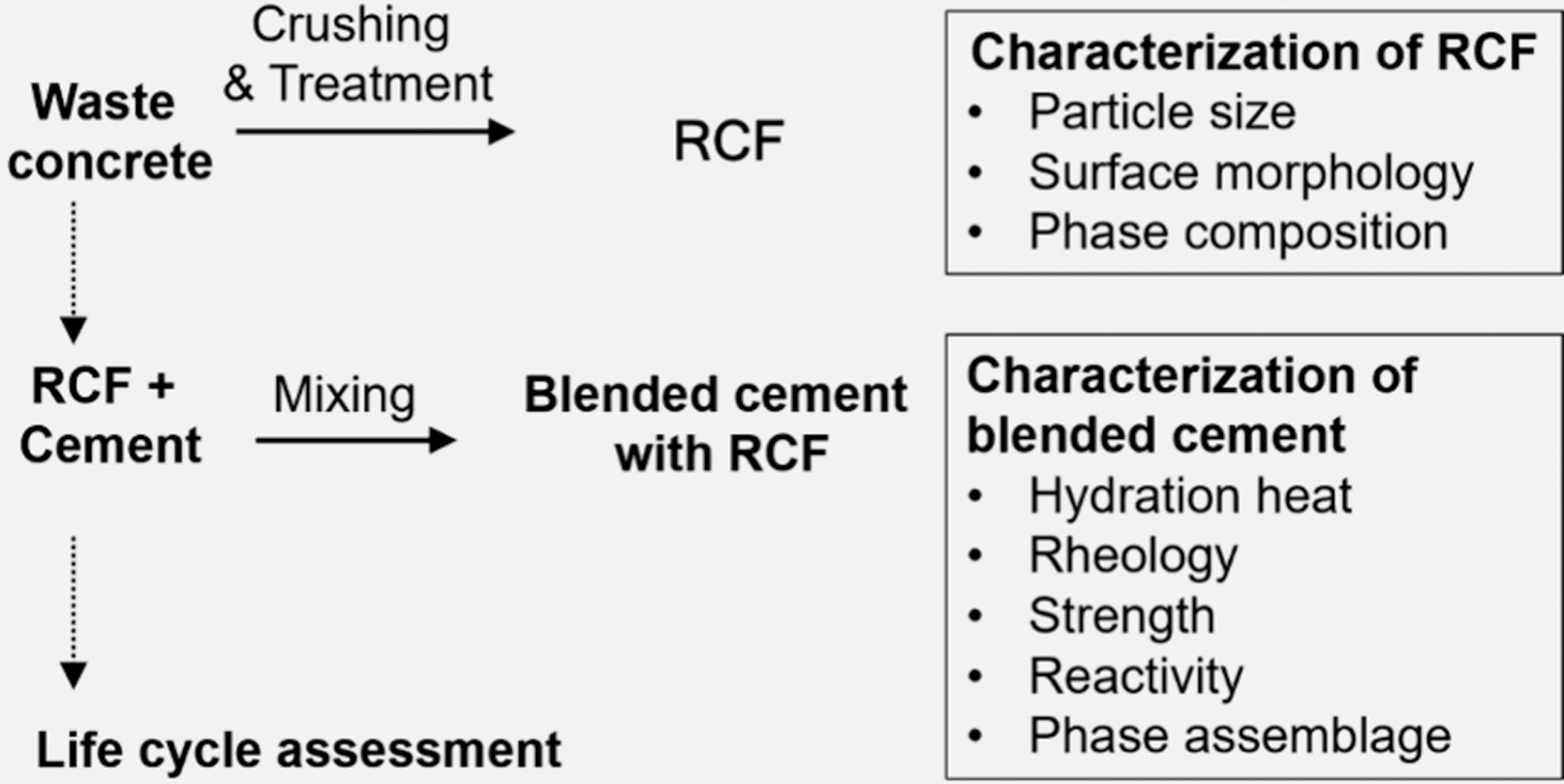
Experimental workflow of RCF in blended cement.

### 2.3 Hydration Heat by Semiadiabatic Calorimetry

This study utilized temperature measurements to assess the hydration heat behavior of blended cement paste mixtures.^30^ A thermally insulated polystyrene formwork with multiple sealable cells was used to contain the fresh cement paste for temperature measurement. Each cell, approximately 5 cm deep and 4 cm in diameter, was sealed with a polystyrene cap. Freshly mixed cement paste was immediately poured into each cell, and an embedded PT100 sensor monitored temperature variations over 24 h to track early hydration.

### 2.4 Rheological Measurement

Rheological measurements of cement paste were conducted by using a Brookfield rheometer (Model RSX). The freshly mixed cement paste was transferred to the rheometer approximately 5 min after contact with water. The procedure involved applying a high shear rate of 100 s^–1^ for 30 s (preshear). This was followed by a 30 s rest to allow the paste to reach a uniform state. The shear rate was then increased from 0 to 120 s^–1^ over the first 120 s and decreased to 0 s^–1^ over the next 120 s. Each shear rate was maintained for 10 s to allow the paste to stabilize (Figure 3). The shear stress at each shear rate was averaged over the last 5 s of each interval. The shear stress and shear rate data were then input into the Bingham model to calculate the dynamic yield stress and viscosity. This rheological protocol (preshear, rest, followed by an up-and-down shear rate ramp) was adopted to eliminate mixing and handling-induced history, establish a reproducible initial state, and capture both flow behavior and thixotropic recovery of the cement paste. Similar procedures have been used to characterize dynamic yield stress, plastic viscosity, and thixotropy of cementitious materials in our previous research.

**Figure 3:**
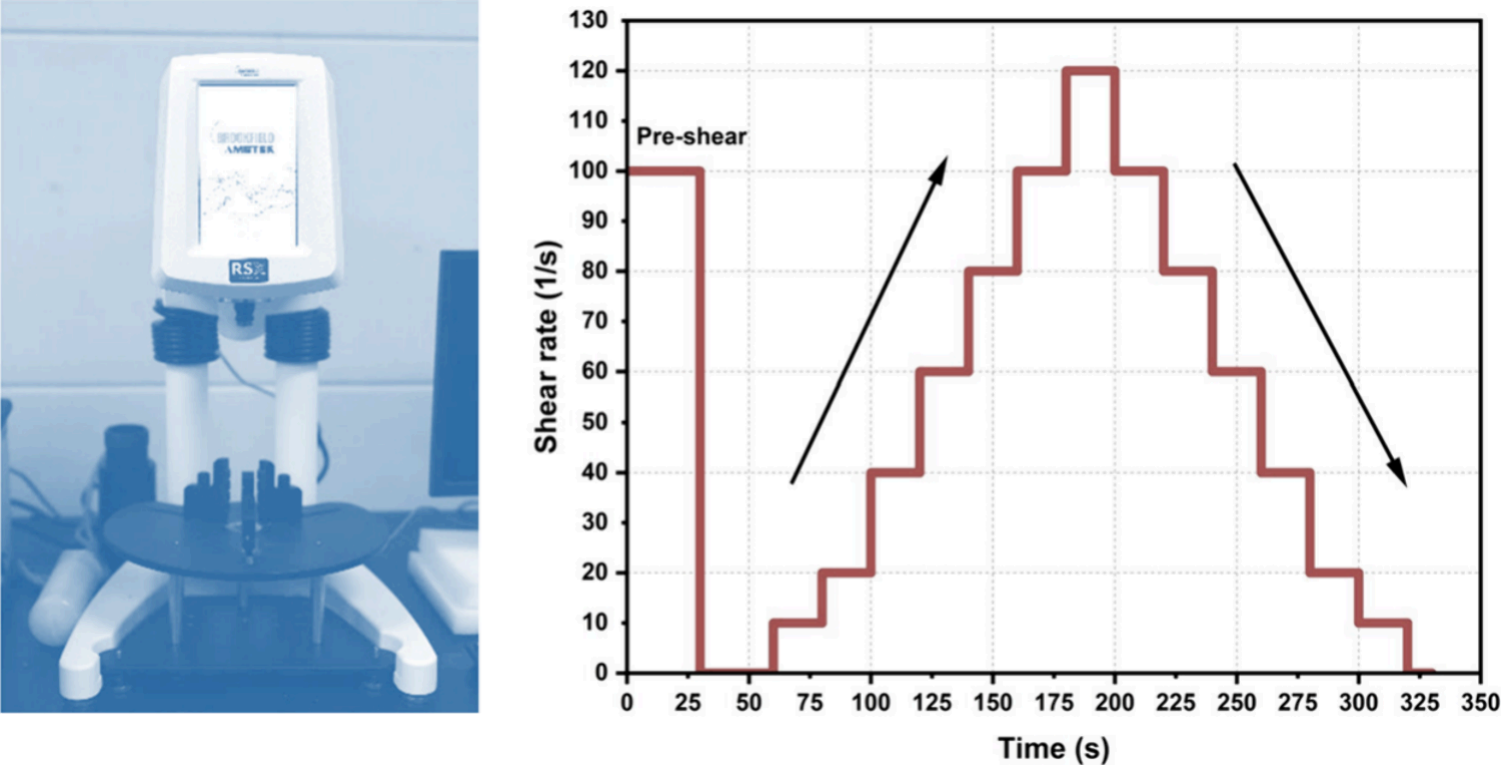
Rheometer used for testing (left) along with the shear rate application procedure during the test (right).

### 2.5 Compressive Strength

Compressive strength was measured by using a universal testing machine with a 100 kN load cell capacity. Testing was performed at a controlled loading rate of 2.4 kN/s until specimen failure. Compressive strength was calculated by dividing the failure load by the cross-sectional area, measured with a caliper. Each result represents the average of three specimens. Specimen weights and dimensions were recorded before testing to determine bulk density.

### 2.6 Microscopic and Phase Analysis

Hydrated cement paste specimens were immediately sampled from the crushed specimens after compressive strength testing. These specimens were then immersed in excess 2-propanol to stop hydration. Afterward, they were placed in a vacuum chamber at room temperature for 24 h to dry. For scanning electron microscopy (SEM) observation, the specimens were fractured to expose fresh surfaces and gold-coated for 240 s. SEM imaging was conducted using a HITACHI TM3000 under high vacuum, with multiple areas examined for a representative understanding of the morphology.

For thermogravimetric analysis (TGA), the hydration-stopped specimens were ground into powder just before testing. Approximately 20 mg of powder was placed in an alumina crucible, and the analysis was performed using a Netzsch STA 449 F3 Jupiter instrument under a nitrogen atmosphere. The temperature was ramped from 25 to 1000 °C at 10 °C/min. For X-ray diffraction (XRD) analysis, the same powder preparation as that for TGA was used. The powder was loaded into a sample holder and compacted with a glass slide. Scanning was conducted using a BRUKER D8 Advance diffractometer with Cu Kα radiation at 40 kV and 40 mA. The scan was performed over a 2θ range of 5°–60° with a step size of 0.03°.

### 2.7 Bound Water Measurement

The bound water measurement, following the ASTM C1897-20 guidelines,^31^ indicates the reactivity of RCF during cement hydration. The model paste, with its mix design detailed in Table 2, was cured in sealed plastic bags at 40 °C for 7 d. The ratio of potassium solution to solids (the sum of RCF, calcium hydroxide, and calcium carbonate) was 1.2 by mass. After being cured, the samples were crushed, sieved through a 2 mm mesh, and oven-dried at 40 °C for 24 h. The dried samples were then dehydrated at 350 °C for 2 h and cooled in a desiccator for 1 h, following the temperature protocol specified in ASTM C1897-20 for bound water determination.^31^ The bound water content was calculated using eq 1:

$$\mathrm{bound}\;\mathrm{water}=\frac{w_{0}-w_{h}}{w_{0}-w_{c}}\times 100$$ 1

where *w*_0_ is the total mass of the dried paste and crucible, *w*_h_ is the total mass of the 350 °C dehydrated paste and crucible, and *w*_c_ is the mass of the empty crucible.

**Table 2:** Mass Proportions of the Model Paste for the Reactivity Test

ingredient	RCF	calcium hydroxide	calcium carbonate	potassium solution^a^
mass (g)	10.0	30.0	5.0	54.0

a The potassium solution consists of 4 g/L KOH and 20 g/L of K_2_SO_4_ dissolved in deionized water.

### 2.8 Life Cycle Assessment

LCA evaluates carbon emissions and potential environmental benefits of incorporating RCF into cement. The study compared blended cement pastes containing RCF with a functional unit of 1 m^3^, including pure cement paste and blended cement pastes with fly ash and limestone powder, as references. Figure 4 shows the system boundaries, covering processes from raw material extraction, processing, and transportation to cement paste production in a ready-mix plant. To reflect realistic conditions, the RCF, cement, limestone powder, and fly ash were sourced from locations near the ready-mix plant. Transportation distances were determined based on these locations.

**Figure 4:**
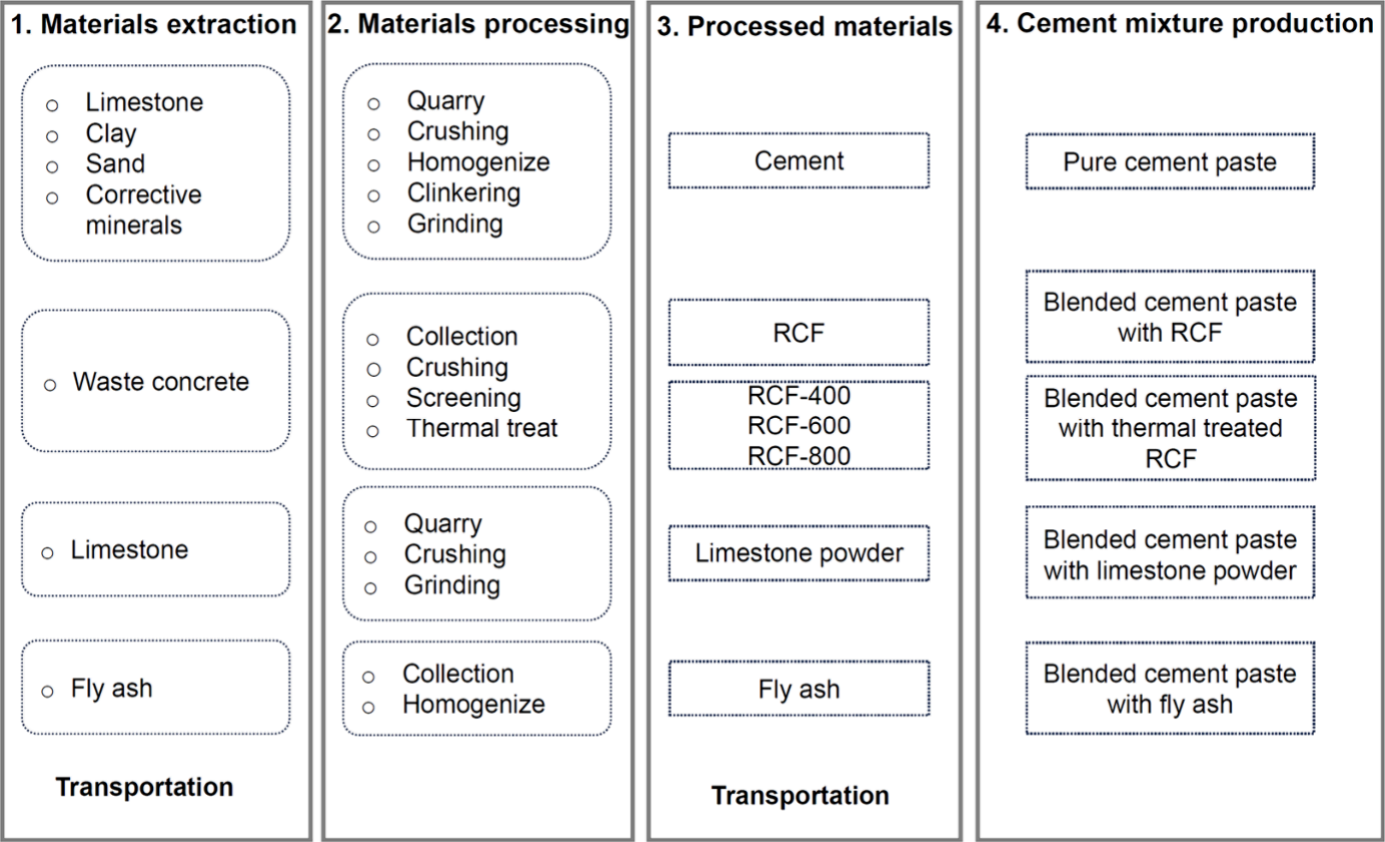
System boundary and process flowchart for blended cement pastes, covering raw material extraction, processing, transportation, and preparation at the ready-mix plant

To enhance transparency, the primary assumptions underlying the LCA are summarized below. Electricity consumption during thermal treatment was calculated from the rated input power of the 15 kg capacity laboratory muffle furnace and the measured treatment time. The resulting electricity use was converted to emissions using a coal-dominated grid mix with an emission factor of 0.94 kg CO_2_-eq/kWh.^32^ The transportation of cement, limestone powder, fly ash, and RCF was assumed to be performed using standard trucks, with the specific distances for each material taken from Table 3. Furnace energy consumption was normalized per unit mass of thermally treated RCF, yielding carbon intensities of 0.2038, 0.3058, and 0.4495 kg of CO_2_-eq/kg for the 400 °C, 600 °C, and 800 °C treatments, respectively. Background data for electricity, transport, and raw material production were obtained from the existing databases and research publications. Total carbon emissions for each paste formulation were then calculated using eqs 2–5, incorporating the carbon intensities for material extraction, processing, transportation, and thermal treatment summarized in Table 4.

$$\mathrm{emission}\;\mathrm{of}\;\mathrm{extraction}+\mathrm{manufacturing}/\mathrm{processing}\;\mathrm{phase}\left(\mathrm{kg}\;{\mathrm{CO}}_{2}-\mathrm{eq}\right)=C_{i}\times 0.8198+{\mathrm{RCF}}_{i}\times \left(0.0006+0.00008\right)+{\mathrm{TRCF}}_{i}\times 0.00068+{\mathrm{LS}}_{i}\times 0.0278+{\mathrm{FA}}_{i}\times 0.0110+W_{i}\times 0.018$$ 2

$$\mathrm{emission}\;\mathrm{of}\;\mathrm{thermal}\;\mathrm{treatment}\;\mathrm{phase}\left(\mathrm{kg}\;{\mathrm{CO}}_{2}-\mathrm{eq}\right)={\mathrm{TRCF}}_{i}\times 0.20384/0.30575/0.4495$$ 3

$$\mathrm{emission}\;\mathrm{of}\;\mathrm{transportation}\;\mathrm{phase}\left(\mathrm{kg}\;CO_{2}-\mathrm{eq}\right)=C_{i}\times 0.0338+{\mathrm{RCF}}_{i}\times 0.0193+{\mathrm{TRCF}}_{i}\times 0.0193+{\mathrm{LS}}_{i}\times 0.3969+{\mathrm{FA}}_{i}\times 0.5287+W_{i}\times 0$$ 4

$$\mathrm{total}\;\mathrm{emission}\left(\mathrm{kg}\;{\mathrm{CO}}_{2}-\mathrm{eq}\right)=\mathrm{emission}\;\mathrm{of}\;\mathrm{extraction}+\mathrm{manufacturing}/\mathrm{process}\;\mathrm{phase}+\mathrm{thermal}\;\mathrm{treatment}\;\mathrm{phase}+\mathrm{transportation}\;\mathrm{phase}$$ 5

where C*_i_*, RCF*_i_*, TRCF*_i_*, LS*_i_*, FA*_i_*, and W*_i_* represent the content of cement, untreated RCF, thermally treated RCF, limestone powder, fly ash, and water, respectively, in each mixture expressed in kg/m^3^ of cement paste.

**Table 3:** Transportation Distances of Raw Materials from Their Sources to the Ready-Mix Plant

raw materials	manufacturing/processing site	ready-mix plant	start-end locations	distance (km)
cement	Taicang	Suzhou	Taicang-Suzhou	70
RCF	Suzhou	Suzhou	Suzhou–Suzhou	40
thermally treated RCF	Suzhou	Suzhou	Suzhou–Suzhou	40
limestone powder	Zibo	Suzhou	Zibo-Suzhou	822
fly ash	Shijiazhuang	Suzhou	Shijiazhuang-Suzhou	1095

**Table 4:** Carbon Intensity of Raw Materials for Extraction, Processing, Transportation, and Thermal Treatment Processes in the Blended Cement Pastes

raw materials	carbon intensity, manufacturing (kg CO_2_-eq/kg)	carbon intensity, transportation (kg CO_2_-eq/kg/km)	carbon intensity, thermal treatment (kg CO_2_-eq/kg)
cement	0.8198 ^33^	0.000483 ^34^	0
RCF	crushing: 0.00060 and screening: 0.00008 ^35^		0
thermal-treated RCF			0.20384 (400 °C)
			0.30575 (600 °C)
			0.4495 (800 °C)
limestone powder	0.0278 ^33^		0
fly ash	0.0110 ^33^		0
water	0.018 ^34^		0

## 3 Results and Discussion

### 3.1 Properties of Recycled Concrete Fines

Figure 5a shows the particle size distribution of the OPC and RCF. The RCF400, RCF600, and RCF800 refer to RCFs thermally treated at 400 °C, 600 °C, and 800 °C, respectively. All RCFs exhibited coarser particle sizes compared to OPC. The untreated RCF displayed a particle size similar to that of RCF400, indicating a minimal particle change at the lower treatment temperature. RCF600 showed a slight reduction in particle size, suggesting fragmentation and disintegration of particles during the treatment. RCF800 exhibited noticeably coarser particles, which may result from particle agglomeration or sintering induced by the higher treatment temperature.

**Figure 5:**
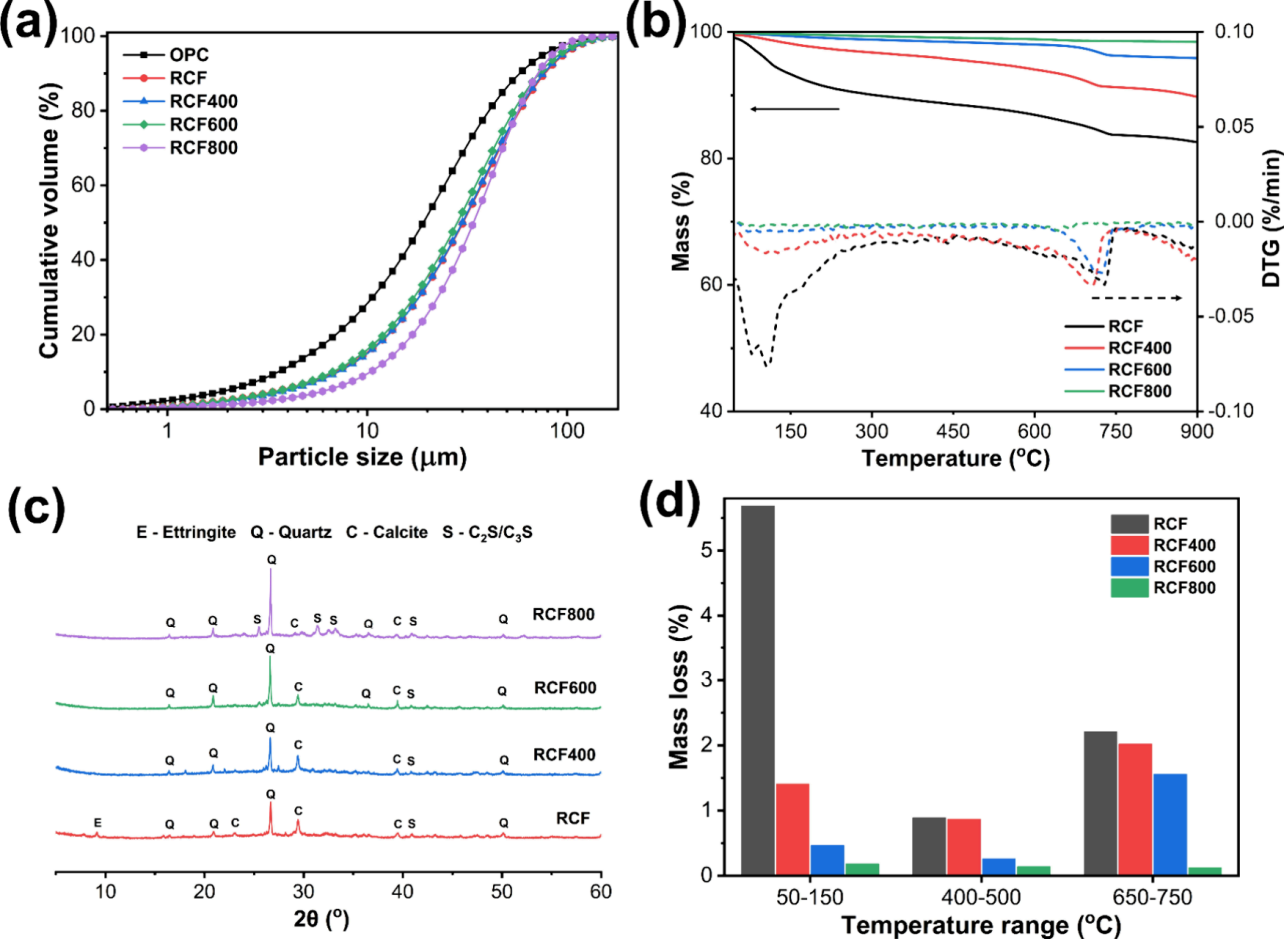
(a) Particle size distribution of OPC and recycled concrete fines (RCF) before and after thermal treatment at 400 °C, 600 °C, and 800 °C; (b) mass loss curves (solid lines, left *y*-axis) and corresponding DTG curves (dashed lines, right *y*-axis) for untreated and thermally treated RCF; (c) X-ray diffraction (XRD) patterns of RCF; and (d) quantified weight loss within temperature ranges of 50–150 °C, 400–500 °C, and 650–750 °C.

Figure 5c shows the XRD spectra of untreated and thermally treated recycled concrete fines. The untreated RCF primarily consisted of quartz, originating from sand debris^36^ and hydrated phases such as calcite and ettringite. After treatment, ettringite was no longer detected, as it decomposed, indicating structural breakdown and enhanced surface reactivity. Portlandite was also not detected, possibly due to carbonation after exposure to air.^37^ After treatment at 600 °C, calcite peaks weakened, suggesting a partial decomposition that may reverse upon cooling, contributing to reactivity. At 800 °C, calcite peaks further diminished, while calcium silicate phases such as C_2_S and C_3_S-like compounds, including wollastonite, emerged, indicating profound dehydration and phase transformation.^38^ The stronger calcium silicate phase peak at 800 °C compared to that at 600 °C suggested that 600 °C may represent an optimal temperature, balancing activation with lower CO_2_ emissions. Besides that, the thermal treatment may also transform C–S–H gel into an amorphous meso-phase structure,^39^ enhancing reactivity. These results indicated that thermally treated RCF contained property-enhanced calcium- and silica-based compounds (e.g., quartz α–β transformation) with potential pozzolanic and filler functions.

Figure 5b,d shows the TGA results of the RCF, which were generally consistent with the XRD findings. The DTG peaks show that at 400 °C, only partial dehydration of C–S–H and ettringite occurs, leaving a significant fraction of stable hydrates and carbonates. At 600 °C, the C–S–H and portlandite decomposition peaks become more pronounced, while carbonate decomposition remains incomplete, indicating that a good portion of CaCO_3_ is preserved. At 800 °C, nearly complete decarbonation and extensive dehydration are observed. Thus, 600 °C corresponds to a kinetic window where weak hydrates are decomposed and more active Ca–Si phases form, while a good carbonate fraction is retained; this balance enhances reactivity and nucleation while avoiding the overcalcination and sintering observed at 800 °C.

Figure 6 shows the surface morphology of untreated and thermally treated RCF. The untreated RCF displayed characteristics typical of a hydrated cement matrix with embedded supplementary cementitious materials and fine filler particles. The surface texture appears complex, suggesting higher water absorption and weaker bonding with cement phases, as observed in the RCF specimen at low, medium, and high magnifications. After treatment at 400 °C, partial disintegration and dehydration were observed, resulting in more fragmented particles in the RCF400 specimen. At 600 °C, further dehydration resulted in more individual particles with less complex surfaces and visible cracking, indicating possible phase changes, such as quartz transformation, as observed in the RCF600 specimen. Following treatment at 800 °C, extensive decomposition occurred. However, some disintegrated particles appeared to recombine, forming secondary agglomerates that reduced overall particle dispersion,^40^ as shown in the RCF800 specimen.

**Figure 6:**
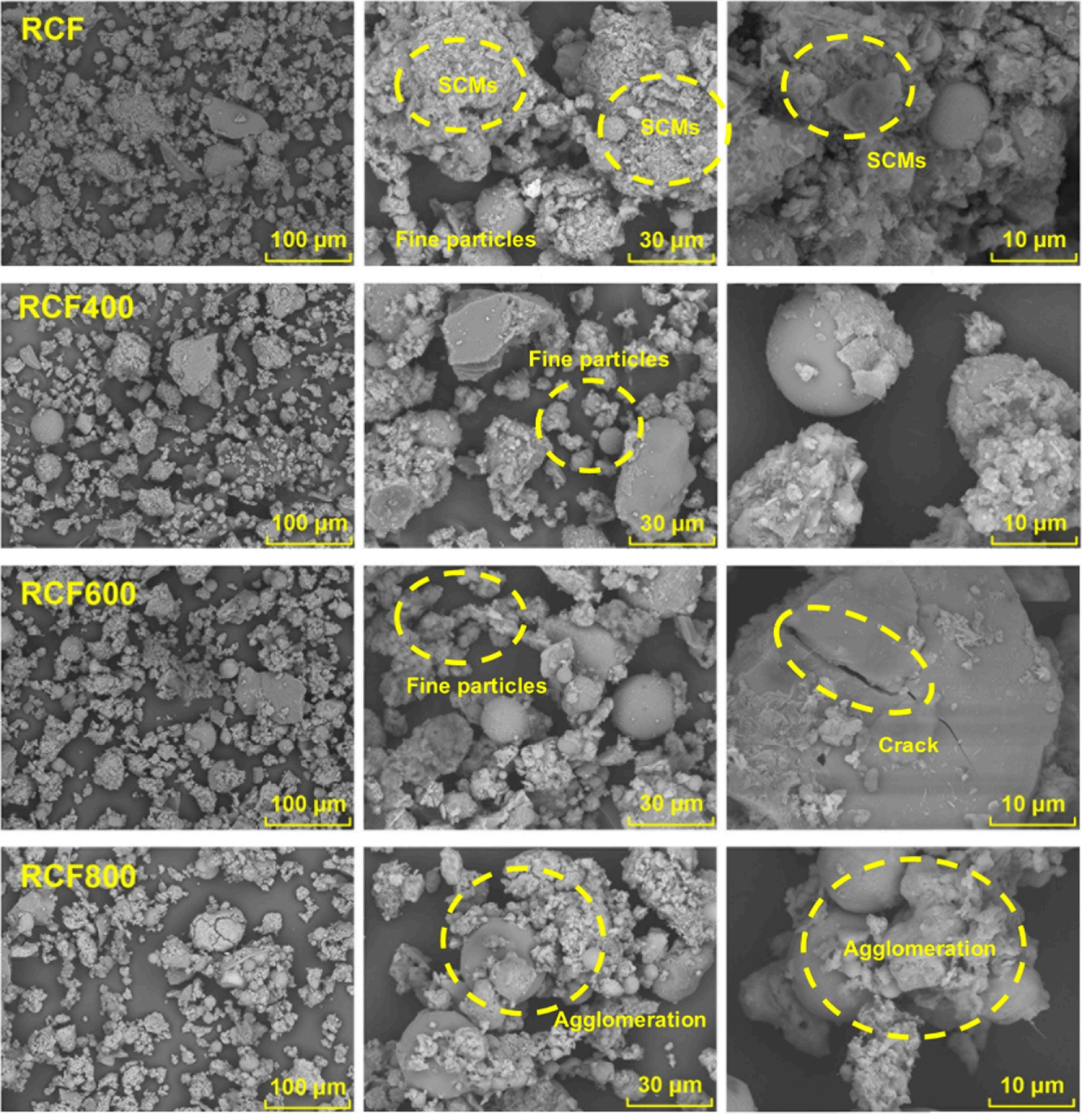
SEM images of untreated (RCF) and thermally treated RCFs at 400, 600, and 800 °C (RCF400, RCF600, and RCF800), showing (left column) lower magnification views to illustrate overall particle distribution, (middle column) medium magnification images highlighting particle clustering, and (right column) high magnification images revealing detailed surface morphology of representative particles.

### 3.2 Properties of Blended Cement with Recycled Concrete Fines

#### 3.2.1 Temperature Profile

Figure 7 shows the temperature of the hydrating cement pastes over 24 h. In the initial hydration stage (Figure 7b), all RCF-blended pastes, whether untreated or thermally treated, exhibited initial temperatures higher than that of the pure cement paste. This rise was primarily attributed to the wetting and water adsorption on the more textured and porous surfaces of RCF particles, which released heat.^41^ Among them, treated RCFs resulted in even higher initial temperatures, suggesting that thermal treatment enhanced the surface reactivity, promoting more intensive interactions with water beyond simple wetting.^28^

**Figure 7:**
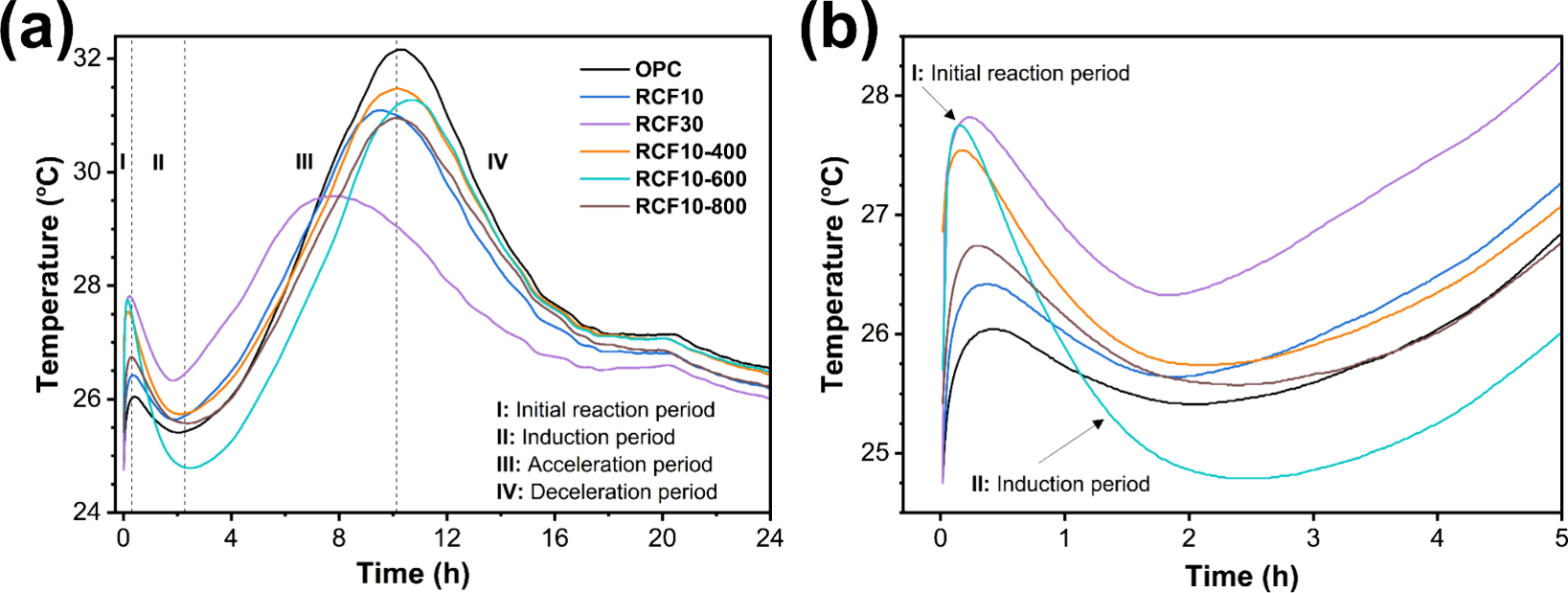
Hydration temperature profiles of blended cement pastes over 24 h: (a) full 24 h monitoring and (b) magnified view of the first 5 h to highlight early hydration behavior.

During the acceleration phase of hydration (Figure 7a), when calcium silicates began to hydrate actively, all RCF-containing pastes showed temperature peaks lower than those of pure cement. This indicated a well-accepted dilution effect from cement replacement and an increase in the effective water-to-cement ratio. Cement pastes with treated RCFs demonstrated greater hydration activity than those with untreated RCFs, confirming the improved reactivity of treated particles. Among the mixtures containing treated RCFs, RCF10–600 exhibited balanced performance considering both the initial and accelerated hydration stages, indicating the roles of its particles and the potential reactivity of its components.

#### 3.2.2 Rheological Properties

Figure 8 shows the rheological properties and thixotropic behavior of fresh cement pastes, including pure cement paste and pastes containing RCF and thermally treated RCF. As the RCF replacement level increased, the dynamic yield stress and viscosity rose. The rougher and more complex surface texture of RCF likely introduced greater interparticle friction and retained more water, reducing the available free water for lubrication. It necessitated more energy to maintain flow, thus increasing both dynamic yield stress and viscosity.^42^

**Figure 8:**
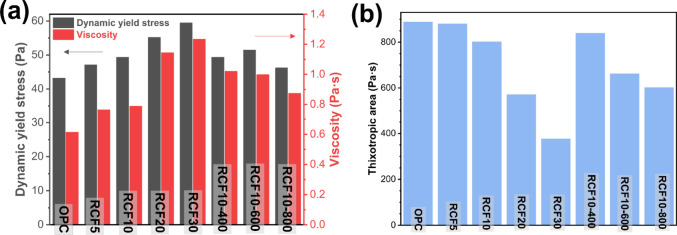
Rheological properties of fresh cement paste: (a) Dynamic yield stress and viscosity of pure cement paste and blended cement paste with untreated and treated recycled concrete fines (RCF) and (b) thixotropic area of pure cement paste and blended cement paste with untreated and treated RCF

Thermal-treated RCF further slightly increased the dynamic yield stress and viscosity of blended cement compared to untreated RCF, although the increase was modest. As the treatment temperature rose from 400 to 600 and 800 °C, the dynamic yield stress and viscosity initially increased and then decreased, consistent with the particle size distribution and phase changes discussed earlier. Regardless of treatment, all RCF-containing mixtures exhibited a higher yield stress and viscosity than the pure cement paste, highlighting the physical (surface roughness and particle size distribution) and chemical (reactivity changes induced by treatment) contributions of RCFs.

The thixotropic area, calculated from the enclosed area between the upward and downward shear stress–shear rate curves, exhibited an opposite trend to yield stress and viscosity. As the RCF replacement ratio increased, the thixotropic area decreased relative to pure cement paste, indicating that while RCFs increased viscosity and yield stress, they did not enhance rigid network formation during early hydration, which was critical for thixotropic recovery.^43^ Upon thermal treatment, the thixotropic area initially increased but slightly decreased with higher treatment temperatures. Thermal modification potentially enhanced the RCF reactivity and promoted stronger network formation. However, higher-temperature-treated RCF (600 and 800 °C) showed reduced thixotropic recovery compared to 400 °C treated RCF, likely due to decreased water absorption capacity. Less water adsorption might result in more water being available in the medium during the initial hydration period, weakening the material’s ability to recover after shear.^44^

#### 3.2.3 Compressive Strength

Since bulk density reflects the internal compactness of hardened cement paste, Figure 9 presents the bulk density and compressive strength of cement pastes containing varying amounts of RCF. As the replacement level of untreated RCF increased (5%, 10%, 20%, and 30%), bulk density consistently decreased. This reduction was attributed to the porosity and reduced hydration products in the matrix, resulting in a less dense microstructure. For all mixes, the bulk density slightly decreased from 3 to 28 days of curing, likely due to gradual moisture loss. This also appeared to influence strength development between 7 and 28 d, suggesting that moist curing with relatively higher humidity is preferred during the early hydration period.^45^

**Figure 9:**
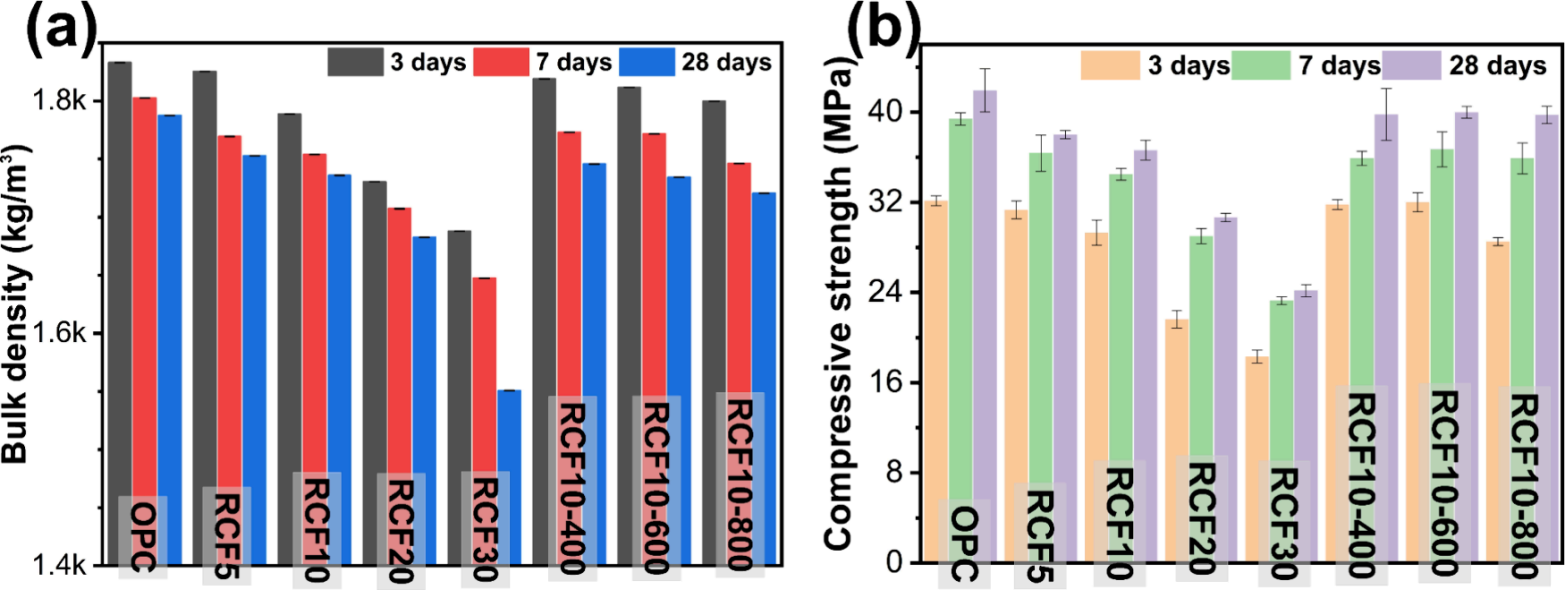
(a) Bulk density of hardened cement paste specimens measured before compressive strength testing and (b) compressive strength results of the corresponding specimens.

When 10% of cement was replaced with thermally treated RCF, the bulk density increased relative to the mixture with untreated RCF at the same dosage. It suggested that thermal treatment improved particle characteristics, such as surface morphology and reactivity, leading to a denser packing and more efficient hydration. As the treatment temperature increased from 400 to 800 °C, the bulk density showed a slight decline, possibly due to sintering of particles or the formation of less reactive phases at higher temperatures.

Compressive strength followed a similar trend. Increasing the untreated RCF content led to a reduction in strength across all curing ages. In contrast, pastes with 10% thermally treated RCF achieved a higher strength than their untreated counterparts. Among the treated samples, RCF calcined at 600 °C yielded the highest compressive strength, indicating an optimal particle distribution, surface activation, and reactivity at this temperature. Strength slightly declined at 800 °C, likely due to particle sintering, which may have altered the particle size distribution and reduced the surface reactivity.

#### 3.2.4 Thermogravimetric, Reactivity, and Surface Morphology Characterization

Figure 10 shows thermogravimetric behaviors for 28 days of hydrated pure cement and blended cement pastes with 10% untreated and thermally treated RCF at 400 °C, 600 °C, and 800 °C. In the temperature range 50–150 °C, the weight loss was primarily due to the decomposition of C–S–H, AFt, and AFm phases. As expected, the pure cement paste exhibited the highest weight loss since it contained the highest amount of these hydration products. The RCF10 specimen showed a slight reduction compared to pure cement, likely because 10% of the cement was replaced by RCF, which also contained its old hydrated phases. The RCF10–400 specimen showed an even lower weight loss, indicating that the old hydration products in RCF were partially decomposed at 400 °C, and the rehydration capacity of the treated RCF was relatively low. The RCF10–600 specimen exhibited an apparent increase in weight loss, comparable to RCF10, indicating that the RCF treated at 600 °C regained some rehydration capacity. The RCF10–800 specimen showed significantly lower weight loss in this range, suggesting that sintering the RCF at 800 °C reduced the rehydration capacity due to the overcalcination and diminished surface reactivity.

**Figure 10:**
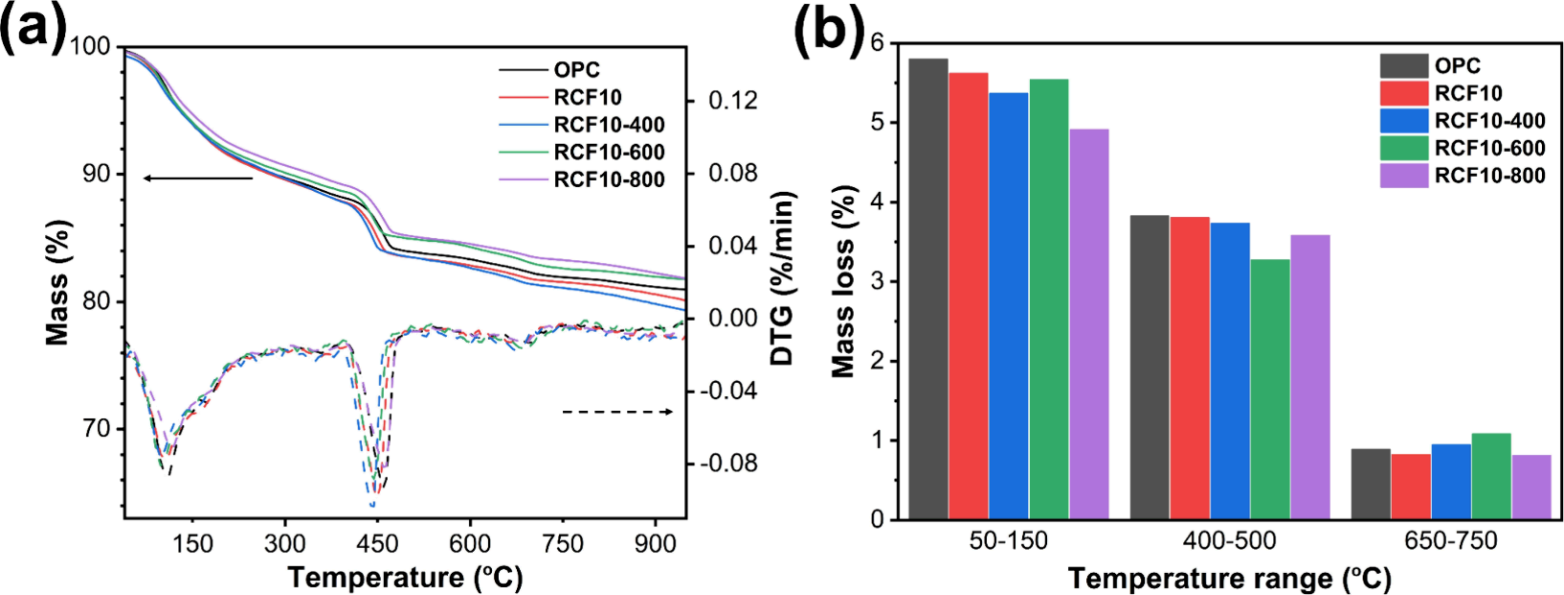
(a) Thermogravimetric mass loss curves (solid lines, left *y*-axis) and DTG curves (dashed lines, right *y*-axis) of hydrated cement paste specimens, indicating phases in hydrated cement paste specimens, and (b) quantified weight loss in the temperature ranges of 50–150 °C, 400–500 °C, and 650–750 °C, corresponding to C–S–H, ettringite, and AFm phases, as well as portlandite and carbonate phases, respectively.

The temperature range 400–500 °C mainly reflected the portlandite decomposition. The weight loss in the pure cement, RCF10, and RCF10–400 specimens was comparable, indicating similar amounts of portlandite. This was reasonable as the blended cement mixtures contained untouched portlandite from the RCF addition (from old cement paste). In the RCF10–600 specimen, the portlandite content was notably reduced, likely due to the reformed calcium hydroxide in RCF600 reacting with other phases in the system such as quartz. This interaction likely contributed to the formation of new hydration products, including C–S–H phases. In the RCF10–800 specimen, the portlandite content was comparable to those of other specimens, indicating that high-temperature sintering affected surface reactivity and hydration. As a result, portlandite levels were higher than those in the RCF10–600 specimen.

In the 650–750 °C range, the carbonate contents of pure cement and RCF10 were similar. However, the carbonate content in the RCF10–600-blended cement specimen was higher than those in all other mixtures. This increase was likely due to the formation of calcium carbonate from the carbonation of calcium-based minerals such as calcium hydroxide, which was more pronounced when RCF was treated at 600 °C. In the RCF10–400 specimen, the carbonate content was also elevated, but to a lesser extent than in the RCF10–600 sample, likely due to the fewer newly formed calcium-based minerals and reduced reactive sites after thermal treatment. At 600 °C, RCF underwent complete decomposition of portlandite and partial decarbonation along with the transformation of some mineral phases, which increased its surface reactivity, facilitating a higher carbonation rate. Conversely, RCF treated at 800 °C experienced greater sintering, which reduced its reaction potential. This limited its carbonation and rehydration capacity in blended cement, leading to less carbonate formation than 600 °C treated RCF.

As shown in Figure 11, the reactivity test results for RCF indicate that the bound water content per 100 g of dry paste was the lowest for the untreated RCF. However, for the specimens with thermally treated RCF, the bound water content increased with the treatment temperature. The bound water content rose progressively from the 400 °C treated RCF to the 600 °C treated RCF and further to the 800 °C treated one. This trend suggested that thermal treatment enhanced the reactivity of the RCF. As the treatment temperature increased, reactivity improved; however, at 800 °C, particle sintering affected reactivity from a particle physics perspective.

**Figure 11:**
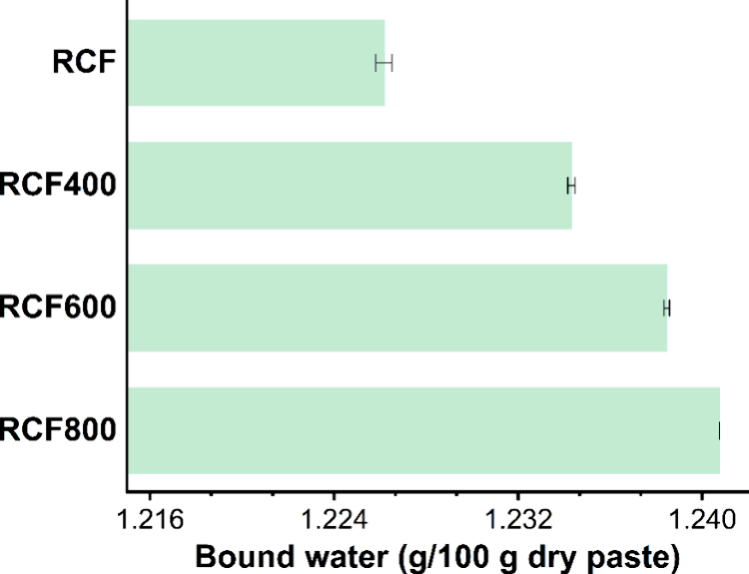
Bound water measurements of cementitious paste containing untreated and thermally treated RCF, indicating the reactivity of RCF, following the standard test methods for measuring the reactivity of mineral additions.

Thermal treatment, surface morphology, and particle distribution influence the reactivity of the RCF in the blended cement. The 600 °C treated RCF performed the best overall in the blended cement, which can be attributed to the optimal balance of surface reactivity and particle distribution achieved at this temperature. The modified surface morphology, particle size distribution, and chemistry of the RCF treated at 600 °C enhanced its integration and interaction with the cement matrix, resulting in better performance than that of the RCF treated at 800 °C.

Figure 12 shows SEM images of hydrated cement cured for 3 and 28 days, including pure cement and blended cement pastes with untreated and thermally treated RCF. The microstructural trends observed aligned well with other measured results. The matrix containing untreated RCF (RCF10) exhibited noticeable porosity and discontinuities between hydration clusters, unhydrated particles, and different phases. Matrices with treated RCF, especially those treated at 400 and 600 °C (RCF10-400 and RCF10-600), showed denser and more cohesive microstructures, similar to the pure cement paste (OPC). The RCF10-600 specimen appeared compact, with clearer bonding between hydration clusters. The RCF10-800 specimen exhibited fewer hydration products and a more open structure, likely due to particle sintering and reduced surface reactivity in the RCF. From 3 to 28 d, while the general morphology of hydration products did not change drastically, an increased presence of the C–S–H network was evident in all samples, especially in the OPC and RCF10-600 specimens.

**Figure 12:**
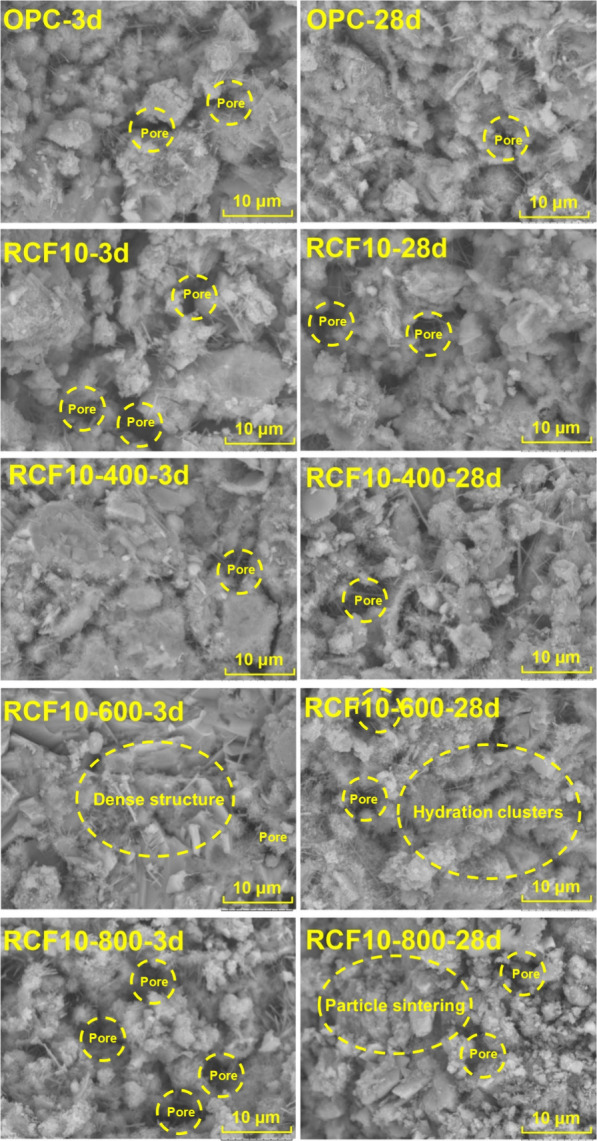
SEM images showing the microscopic surface morphology of hydrated cement paste specimens cured for 3 days (left column) and 28 days (right column). The specimens include pure cement paste (OPC) and blended pastes with untreated RCF and thermally treated RCF at 400 °C, 600 °C, and 800 °C (RCF10, RCF10-400, RCF10-600, and RCF10-800, respectively).

The SEM images also suggest changes in the integration of RCF particles into the blended cement matrix, indicating the quality of the interfacial transition zone. For example, in RCF10 and RCF10-800, microdisintegrations and more porous regions are visible around particles and clusters, whereas RCF10-600 exhibits a denser and less distinct disintegration, evidenced by the more consistent and filled matrix with clearly more hydration product enrichments, indicating improved bonding and rehydration at the interface. Such densification is consistent with enhanced mechanical performance and has been correlated with interface enhancement, as reported with microfibers in a concrete matrix.^46^ The weakening of calcite peaks at 600 °C (Figure 5c) and the elevated carbonate content of the RCF10-600 paste in the 650–750 °C range (Figure 10b) indicate partial decarbonation during heating, followed by recarbonation during cooling and subsequent hydration. This sequence likely produces finely dispersed CaCO_3_ and silica gel on the RCF surface, which can act as nucleation sites and contribute to interfacial densification and improved bonding. A similar mechanism has also been reported in a recent study.^47^ To complement SEM observations, a 2D porosity quantification was conducted using ImageJ by threshold-segmenting the pore regions in the 28 d images. The resulting porosities were 16.9% for the OPC, 25.6% for RCF10, 24.8% for RCF10-400, 17.0% for RCF10-600, and 25.2% for RCF10-800. These results confirm that the RCF10-600 matrix develops a more compact microstructure among all RCF-blended pastes, consistent with its improved strength and hydration behavior. In contrast, untreated RCF10 and RCF10-800 retain clearly higher pore fractions, which is consistent with their less compact morphology and lower reactivity. Similar findings regarding the microstructure have also been reported in a recent study.^48^

#### 3.2.5 Carbon Emissions

In Figure 13, the carbon emission for each cement paste is shown and calculated using the emission factors in Table 4 and mixture compositions in Table 1 and eqs 2–5. Figure 13a presents the carbon emissions of pure cement paste, blended cement paste incorporating untreated and thermally treated RCF, and reference mixtures containing limestone powder and fly ash. The emissions are expressed in kilograms of CO_2_-eq per m^3^ of cement paste. An enlarged view in Figure 13b highlights detailed comparisons among blended cement pastes with 10% cement replacement by untreated RCF and RCF treated at 400 °C, 600 °C, and 800 °C, alongside fly ash and limestone powder. The comparison provides insight into the carbon footprint of RCF relative to that of traditional cement replacements. The total carbon emission results showed that replacing 10% of cement with RCF, limestone powder, or fly ash reduced emissions by approximately 9.7%, 5.0%, and 3.6%, respectively. RCF offered advantages due to its local availability, effectively eliminating transportation-related emissions, unlike limestone powder and fly ash, which often faced sourcing and availability challenges. Limestone powder had a lower transportation footprint than fly ash, but its production seemed to be more emission-intensive.

**Figure 13:**
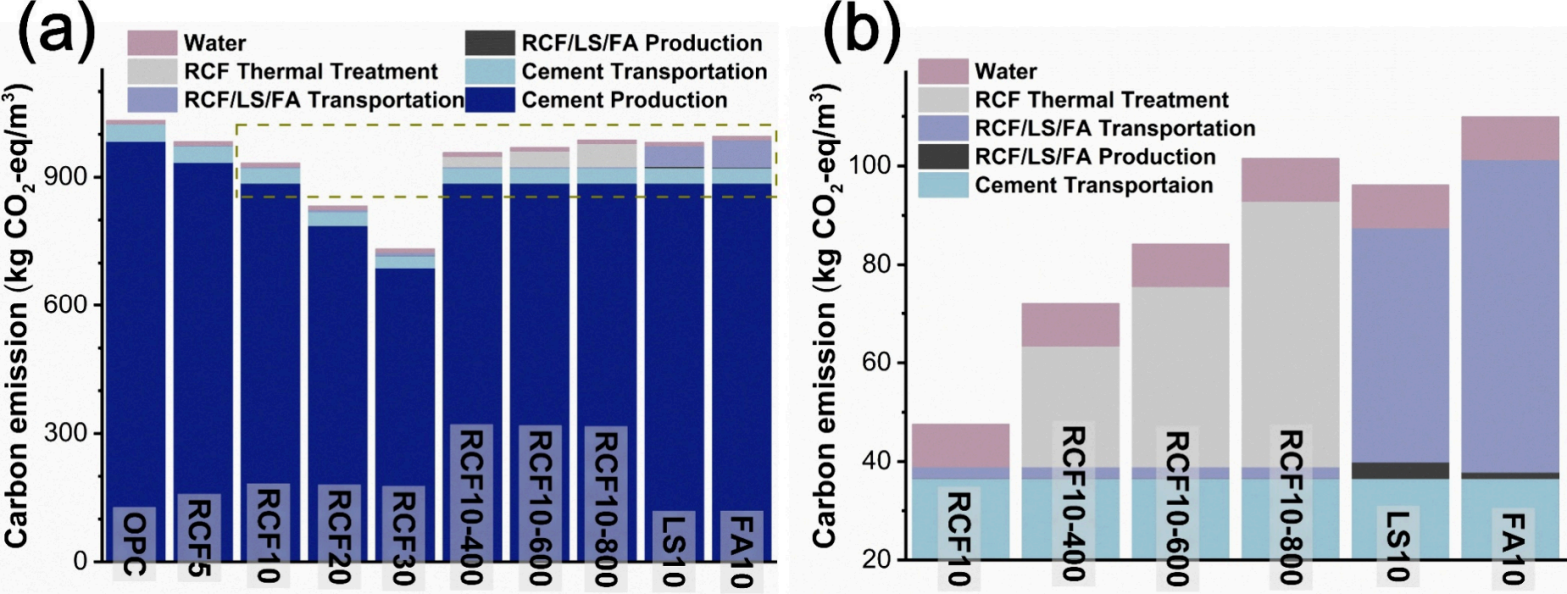
(a) Carbon emissions of pure cement paste, blended cement pastes with untreated and thermally treated RCF, and reference cement blends with limestone powder and fly ash, and (b) enlarged view highlighting detailed comparisons among blended cement pastes containing 10% RCF (untreated and treated at 400 °C, 600 °C, and 800 °C), fly ash, and limestone powder. Carbon emissions are reported in kg CO_2_-eq/m^3^ of cement paste.

During the production and processing phase, the CO_2_ emissions of RCF were roughly three times lower than those of limestone powder and about half of those from fly ash. This was attributed to RCF’s straightforward processing, mainly crushing and screening, which consumed significantly less energy compared to the mining of limestone and the collection and processing of fly ash. Although thermal treatment of RCF introduced additional emissions due to heating, its overall carbon footprint remained lower than those of limestone powder and fly ash, primarily due to savings in transportation emissions thanks to its local availability. Compared to pure cement, total carbon emissions were reduced by 8.8%, 8.3%, 7.7%, 5.0%, and 3.6% for RCF10-400, RCF10-600, RCF10-800, LS10 (limestone powder blend), and FA10 (fly ash blend), respectively. RCF treated at 600 °C demonstrated the best balance of performance and sustainability in this work, highlighting its potential as a viable recycled mineral additive for cement. At 28 d, the RCF10-600 mixture retained 95% of the OPC compressive strength, which was 0.5 and 0.6 percentage points higher than RCF10-400 and RCF10-800, respectively, while still achieving a 6.1% reduction in CO_2_ emissions relative to OPC (Figures 9 and 13). When normalized by carbon footprint, RCF10-600 provided the highest ratio of compressive strength per unit of CO_2_ (MPa kg^–1^ CO_2_-eq) among all RCF-containing mixtures, indicating the most favorable balance between mechanical performance and environmental impact.

A brief scenario analysis was conducted to assess the robustness of these results under different energy sources and logistical conditions. Two common representative variations were considered: (i) a lower-carbon electricity mix in which the grid emission factor was reduced by 30% via the incorporation of renewable energy into the grid and (ii) an adverse transport scenario in which the transport distance of limestone powder and fly ash was doubled while RCF remained locally sourced. Under both scenarios, absolute emission values changed as expected but the relative ordering of mixtures remained unchanged. In particular, the RCF10-600 mixture consistently provided the most favorable combination of reduced CO_2_ emissions and high compressive strength, confirming that the conclusions of this study are not sensitive to reasonable variations in electricity generation or transport assumptions.

To further support 600 °C as the optimal treatment temperature, two quantitative indicators were evaluated. First, the 28 day strength gain per 100 °C increment was calculated. Between 400 and 600 °C, the compressive strength increased from 39.793 to 39.983 MPa, corresponding to +0.095 MPa per 100 °C. In contrast, heating from 600 to 800 °C resulted in a decrease from 39.983 to 39.750 MPa (−0.233 MPa) or −0.117 MPa per 100 °C. These results indicate that meaningful strength enhancement occurs only within the 400–600 °C interval. Also, an environmental efficiency measure was introduced by comparing the incremental carbon emissions with the corresponding strength change. The CO_2_ emissions of the RCF10 mixtures were 957.39, 969.62, and 986.87 kg CO_2_-eq/m^3^ for the 400 °C, 600 °C, and 800 °C treatments, respectively. Moving from 400 to 600 °C increased emissions by 12.2 kg CO_2_-eq/m^3^ while providing a 0.190 MPa strength gain (64.2 kg CO_2_-eq per MPa). In contrast, the 600–800 °C step increased emissions by 17.3 kg of CO_2_-eq/m^3^ while causing a 0.233 MPa strength loss, confirming that 800 °C offers no clear environmental or mechanical benefit. These quantitative indicators demonstrate that 600 °C provides a favorable balance between strength development and carbon emissions.

### 3.3 Implications

RCF consists mainly of hydrated cement, unhydrated cement particles, and fine sand. Among the different processing methods, thermal treatment appears to be a practical and feasible approach for enhancing RCF reactivity. This strategy is conceptually similar to clinker production but requires significantly lower temperatures and could utilize renewable energy or industrial waste heat. These advantages make it especially promising in regions, such as China, where both large amounts of waste concrete fines and increasing renewable energy availability are present.

With respect to treatment temperature, the findings of this and our previous studies suggest that a medium range around 600 °C is the most effective. At this level, both the hydrated cement and fine sand/filler components of RCF are activated, improving the material’s suitability as a cement substitute. In contrast, lower temperatures (≈450 °C) only partially activate the hydrated cement fraction, leaving the remaining hydrates and the fine sand essentially inert, even though waste concrete fines typically contain considerable amounts of sand. Higher temperatures (≈800 °C) can also achieve activation but tend to cause surface melting and particle agglomeration, which may reduce the effectiveness of the treated RCF when blended into cement.

The observed improvements at 600 °C can be understood within a multiscale framework. At the microscale, thermal treatment destabilizes ettringite and portlandite while partially decarbonating calcite and transforming C–S–H and quartz, generating active calcium- and silica-rich phases (Figures 5 and 10). At the mesoscale, these changes produce a broader particle size distribution and less clustered particles (Figure 6), promoting denser packing and a more continuous C–S–H network in the hardened matrix (Figure 12). These micro- and mesostructural reorganizations translate into macroscale enhancements in hydration kinetics, rheology, and compressive strength, while also lowering cement content and associated carbon emissions. Similar multiscale correlations have been reported in studies of recycled aggregate concrete with advanced 4D CT techniques.^49^

The recycling and treatment of RCF could be integrated into existing concrete recycling and cement manufacturing infrastructures, with relatively minor modifications. By valorizing construction waste and reducing clinker demand, thermally treated RCF has the potential to lower CO_2_ emissions in the cement industry. Further research could explore coupling thermal treatment with mechanical activation to maximize efficiency while minimizing energy input. While this study focused on the fraction of waste fines with particle sizes comparable to cement, future work could extend the scope to particles up to ∼1 mm. This would allow inclusion of a broader range of fines, particularly mortar-derived particles containing both hydrated cement and fine sand, which could first be thermally activated and subsequently milled to sizes comparable to those of cement. Also, this study focused on static compressive strength, but the observed interfacial densification and matrix refinement in the cement matrix containing 600 °C treated RCF suggest potential benefits for toughness and dynamic mechanical behavior. Previous research on high-toughness recycled aggregate concretes has shown that improved interface quality and mesoscale morphology can significantly affect the dynamic response and damage evolution under high-strain-rate loading.^50^ Therefore, expanding the RCF blended cement study to include dynamic mechanical characteristics is an important direction for future research.

Beyond stand-alone thermal activation, the present findings indicate the potential for combining thermally treated RCF with other activation or reinforcement strategies. Mechanical milling, following heat treatment, may further increase the fineness and surface area of the RCF, thereby amplifying its filler effect, nucleation, and surface capacity. Carbonation treatment represents another promising approach, as it can generate dispersed CaCO_3_ and silica-gel reaction layers on the RCF surface, further strengthening the ITZ. Additionally, integrating thermally treated RCF with fibers or microfibers could potentially produce synergistic improvements in matrix densification and microdefect resistance, as observed in composite systems. These possibilities highlight that thermal activation of RCF, like other mineral additives, can be incorporated into multimechanism frameworks, offering diverse pathways for advancing low-carbon, high-performance cementitious materials.

## 4 Conclusions

This study demonstrated that thermal treatment effectively transforms recycled concrete fines (RCFs) into reactive mineral additives for cement. Heating destabilized stable hydration products and induced phase changes in the sand fraction, yielding poorly crystalline and reactive phases with a higher surface activity. These changes broadened the particle size distribution and improved surface morphology, enabling more uniform blending with cement compared to untreated RCF, which suffered from excessive water absorption and poor fresh-state compatibility.

Blended systems incorporating thermally treated RCF exhibited accelerated hydration kinetics, improved rheological behavior, and enhanced compressive strength. Microstructural analyses confirmed denser matrices with increased hydration products, particularly when RCF was treated at 600 °C, which emerged as the optimal activation condition. Life cycle assessment further showed that thermally treated RCF reduced overall CO_2_ emissions relative to limestone powder and fly ash, owing to its local availability and simpler processing.

These results clearly link the treatment temperature, reactivity, and sustainability performance. Thermal treatment at moderate temperatures (∼600 °C) offers a practical pathway to valorize construction waste, while lowering clinker demand and emissions in cement production. Looking forward, coupling thermal activation with mechanical processing, so as to extend the usable fraction of recycled fines to coarser particles (up to ∼1 mm), and integrating renewable or waste heat sources could further enhance the resource efficiency, carbon reduction potential, and industrial feasibility of this approach.
